# Impact of energy density on energy intake in children and adults: a systematic review and meta-analysis of randomized controlled trials

**DOI:** 10.1007/s00394-022-03054-z

**Published:** 2022-12-02

**Authors:** Bea Klos, Jessica Cook, Letizia Crepaz, Alisa Weiland, Stephan Zipfel, Isabelle Mack

**Affiliations:** Internal Medicine VI, Department of Psychosomatic Medicine and Psychotherapy, University Hospital Tübingen, Osianderstr. 5, 72076 Tübingen, Germany

**Keywords:** Energy density, Energy intake, Manipulation, Nutrition, Diet, Obesity

## Abstract

**Purpose:**

The energy density (ED) of a diet can be leveraged to prevent weight gain or treat overweight and obesity. By lowering the ED of the diet, energy intake can be reduced while maintaining portion size. However, a reliable meta-analysis of data from randomized controlled trials (RCTs) is missing. Therefore, this meta-analysis synthesized the evidence of ED manipulation on energy intake in RCTs.

**Methods:**

The systematic literature search of multiple databases according to PRISMA criteria considered RCTs investigating the objectively measured energy intake from meals with different ED (lower ED (median 1.1 kcal/g) versus higher ED (median 1.5 kcal/g)) under controlled conditions. Subgroup analyses for age (children versus adults), meal type (preload versus entrée design), and intervention length (1 meal versus > 1 meal) were performed to achieve the most homogeneous result.

**Results:**

The meta-analysis of 38 included studies demonstrated that lowering ED considerably reduced energy intake – 223 kcal (95% CI: – 259.7, – 186.0) in comparison to the higher ED interventions. As heterogeneity was high among studies, subgroup analyses were conducted. Heterogeneity decreased in subgroup analyses for age and meal type combined, strengthening the results. An extended analysis showed a positive linear relationship between ED and energy intake. Dietary ED did not affect the amount of food intake.

**Conclusion:**

Manipulating ED substantially affects energy intake whereas food intake remains constant. Thus, this approach can be regarded as a powerful tool for weight management through nutrition therapy. Registration on 08/08/2021: CRD42021266653.

**Supplementary Information:**

The online version contains supplementary material available at 10.1007/s00394-022-03054-z.

## Introduction

Body weight maintenance is based on the balance between energy intake and energy expenditure. Consequently, the principles of weight management focus on reducing energy intake by changing diet and eating patterns while increasing energy expenditure through higher levels of physical activity and reduced sedentary behavior [1].

Food portion size and energy density (ED; calories per gram) are critical determinants of energy intake [1, 2], which is why their manipulation is the basis of many weight management interventions [3–6]. Thus, foods with high water content have a low ED, whereas foods containing high proportions of macronutrients, especially carbohydrates and fats, have a higher ED [7]. Many laboratory studies have shown that the reduction of meal ED allows for consumption of a satiating amount of food while simultaneously reducing energy intake [8, 9] due to relatively constant amounts of food consumed across conditions [10, 11]. In response, the World Health Organization has highlighted energy-dense foods as a key contributing factor to the increasing prevalence of overweight and obesity [12], noticing the role of either low- or high-energy-dense food selection in the diets has on body weight [13–16]. In practice, the methods that lower ED are not only flexible and adaptive, but also allow for application in a great variety of dietary patterns, aligning with individual food preferences as well as personal and cultural backgrounds [2, 17, 18].

Although, data on the impact of an ED intervention on energy intake in both children [19] and adults [20] clearly point towards the same outcome, study designs are highly heterogeneous in relation to intervention and duration approaches. First, differing satiation mechanisms are triggered based on either an entrée or a preload design. Preload interventions trigger between-meal-satiety at the end of the preload inhibiting further food intake, whereas, in an *ad* *libitum* entrée design, intra-meal satiation is initiated, triggering meal termination and determining the size of the meal [21]. A systematic review and meta-analysis of clinical trials was performed to assess the effect of preload ED on energy intake in subsequent meals. The analysis revealed that compared to a high ED preload, a low ED preload resulted in higher subsequent energy intake, but the total energy intake (preload + subsequent meal) was still lower in the latter condition [22]. Similarly, participants showed a trend toward some compensation for reduced energy intake after consuming preloads, but without reaching statistical significance [23]. Conversely, when subjects were satiated consuming either high or low ED *ad libitum* entrées for 5 days, the subjects in the low ED group halved their energy intake without any compensation [24]. Second, differences in intervention length contribute to more heterogeneity since the effects of ED manipulation differ in short-to-medium term studies versus longer term interventions [25]. A recent meta-analysis confirmed that manipulating ED for at least one day results in significantly altered energy intake. The meta-analysis of 41 studies with human participants examined randomized and non-randomized experimental studies that took place either in the laboratory or in a free-living setting [19]. Moreover, it has been suggested in an experimental study, that over longer experimental settings the amount of food consumed would gradually increase to compensate for reduced ED and therefore the daily energy intake would be maintained [10].

Currently no reliable meta-analysis of data regarding the impact of ED manipulation on energy intake in humans from exclusively randomized controlled trials (RCTs) considering data of objectively measured food intake exists. Additionally, the relationship between the offered ED of foods and energy intake has not been mathematically described and remains unclear. This systematic review and meta-analysis aimed to close these gaps by synthesizing the best available evidence on the effectiveness of influencing the energy intake by manipulating ED in children and adults. In addition, different meal types and intervention lengths were considered. The secondary objective was to analyze the impact of the various ED conditions on the amount of food intake. The following hypotheses were tested qualitatively and quantitatively:

- 1. Energy intake in kcal is lower in the meal conditions with lower ED in comparison with the meal conditions with higher ED.
- 2. The amount of food in g consumed is similar across the meal conditions with different ED.

## Materials and methods

This review was developed and executed according to the Preferred Reporting Items for Systematic Reviews and Meta-Analyses (PRISMA) guidelines [26]. To identify all relevant studies examining the effect of ED manipulation on the consumed energy in humans the databases PubMed, Web of Science, Cochrane Library (Wiley) and EBM-Reviews (Ovid) Cochrane Library were searched on December 14th 2020 and updated at January 6th 2021. The protocol of this systematic review is registered on the PROSPERO platform with the registration number CRD42021266653. The full search strategy is documented in the supplementary information (Text S1) and consists of the four modules manipulated ED, group comparison, energy intake and the exclusion of animals.

### Eligibility criteria

Inclusion criteria were established based on the five PICOS dimensions, i.e., participants, interventions, comparator, outcome and study design [27].

#### Participants

Human adults, adolescents and children aged older than one year, without any restrictions on sex and weight status were included. Participants unable to eat solid foods were excluded (e.g. breast, complementary or tube feeding). Studies exclusively conducted in specific patient groups with e.g. type 2 diabetes/ insulin resistance, cardiovascular disease, metabolic syndrome, cancer, immunodeficiency diseases, malnutrition/anorexia, renal disease, diarrhea or after any kind of surgical intervention were excluded to avoid selection bias of specific groups. In addition, articles examining participants with food intolerances or food allergies were not considered.

#### Interventions

A controlled environment (such as a laboratory or a researcher manipulated group setting) was required. Participants were required to be served at least one test meal per day with a manipulated ED, resulting in meals with a lower and a higher ED. Studies were included that had either (i) a compulsory manipulated preload (all participants had to consume the same amount) followed by an unmanipulated *ad libitum* test meal, or (ii) solely an *ad libitum* entrée manipulated in the ED, or (iii) a manipulated *ad libitum* entrée and unmanipulated compulsory side dishes (all participants were required to eat the offered side dishes), or (iv) a manipulated *ad* *libitum* entrée and unmanipulated *ad libitum* side dishes. Studies that achieved manipulation of ED either by modifying the proportions of macronutrients in % (e.g., low-fat versus high-fat products) or by changing the water content (e.g. adding water or vegetables), or by substituting foods of a lower or a higher ED (e.g. commercially available food products) were eligible. Studies that altered the ED of meals by increasing the amount of water (ED 0.0 kcal/g) consumed during meals in the form of a beverage were excluded since it was found that water had a greater impact on satiety when included in food than when consumed as beverage along with food [28], as water blended into foods has been shown to slow stomach emptying more than consuming the water separately [29]. Studies focusing on portion size manipulation, conditioning periods for becoming familiar with a certain food or energy intake, studies that allowed alcohol consumption within the intervention or that focused on physical activity were excluded.

#### Comparator

A comparison among intervention arms was required either between or within subjects.

#### Outcome

The primary outcome was energy intake in kcal resulting from the corresponding ED manipulation. Therefore, food intake in g had to be measured by a calibrated scale and the foods’ caloric value had to be derived from validated sources, either bomb calorimetry or internationally known food databases. Data from FFQs, 24-h recalls or similar sources were excluded. The secondary outcome was food intake in g after the meals.

#### Study design

The systematic data analysis referred exclusively to randomized trials as parallel and crossover designs.

### Study selection

To identify eligible studies, the search results of the databases were combined and the duplicates removed. Two authors (B.K. and J.C.) independently screened titles and abstracts to identify relevant randomized trials. Full-text articles were evaluated regarding their eligibility with uncertainties being discussed between the authors (< 3% cases). In case of discrepancies, a third author was involved (I.M.). In case of missing data, the authors of the RCTs were contacted by email with a response rate of 85%.

### Data extraction

The following information was extracted from each included article: year of publication, country of origin, study design and intervention length, sample characteristics (including age, sex, body mass index (BMI) or BMI percentile curves) and sample size, details of the intervention (modified ED per meal, manipulated meal and the type of the manipulated meal, time of day to which the meal belongs (breakfast, lunch, dinner), possible other meal manipulations), outcomes including energy intake in kcal and food intake in g and information for assessment of the risk of bias were recorded.

Characteristics across studies are presented as median [interquartile range], minimum and maximum for sample size, age, BMI or BMI percentile curves, study length, washout period and provided ED. The results across studies are presented as per cent (%) for origin and sex distribution.

### Data analyses

For energy and food intake, data were evaluated qualitatively and quantitatively (meta-analysis). The qualitative analyses allowed all findings to be summarized regarding their direction of change between the intervention groups, as not all studies provided sufficient data. For the meta-analysis, a random-effect model was applied [30] using the software package Review Manager, version 5.4.1 [31] and sample size, energy intake in kcal, food intake in g presented as mean and SD are reported separately for lower ED and higher ED intervention. The difference is expressed as mean difference and 95% confidence interval, and graphically presented in forest plots. To eliminate underweighting and double-counting errors, factorial crossover designs were included in the analysis once per ED intervention according to their factor (2 × 2 or 3 × 2) as recommended by the Cochrane Handbook [32]. Thus, multifactorial studies were recorded as single effects in the quantitative analyses and were separated by a unique study ID. Statistical heterogeneity was examined by visual inspection of forest plots and using the *I*^2^-statistics to quantify inconsistency between the studies. *I*^2^-heterogeneity below 40% was considered as low, whereas heterogeneity up to 60% was classified as moderate and from 75% onwards as considerably high [32].

Subgroups were developed to provide a more homogenous summary of findings. In both the qualitative and quantitative analyses, studies were classified according to age of the study population. Hence, the subgroups of children and adolescents (< 18 years, BMI given by percentiles) and adults (> 18 years, BMI given in kg/m^2^) were identified (subgroup analysis 1). For the quantitative meta-analysis, additionally the subgroups regarding meal type (preload vs. entrée; subgroup analysis 2) were formed. In preload studies, study participants are served a mandatory preload followed by an *ad libitum* test meal. The time between preload and test meal had to be at least 15 min. Only the combined data of preload and subsequent test meal was used for the analysis. In contrast, entrée studies included meals with different ED served *ad libitum*. Side dishes were possible and were considered as well. Finally, the data were analyzed according to intervention length (energy intake after 1 meal is assessed versus energy intake after > 1 meal is assessed; subgroup analysis 3). A statistically significant subgroup effect was considered at a *p* -value of less than 0.05 [32]. No subgroup analysis was performed in the context of weight status as the sample was too small (*n* = 3).

To analyze the relationship between consumed delta ED (lower versus higher ED condition per study) and delta energy intake, linear regression analyses were conducted. Thereby, the consumed ED was calculated from objectively measured energy [kcal] and food intake [g]. The ED consumed was not calculated in situations where data on food intake was absent. Regression analyses were only performed when more than 5 cases were available to avoid any bias, allowing for analyses of the following subgroups: (i) children, entrée design, 1 meal; (ii) adults, entrée design, 1 meal, (iii) adults, entrée design, > 1 meal, (iv) preload design. The coefficient of determination *R*^2^ was used as a criterion for linearity. Generally, higher coefficients indicate a better prediction of the dependent variable, where coefficients of 0.5 or more are considered to indicate a linear relationship [33].

### Risk of bias

For the eligible studies, a risk of bias assessment was conducted using the Cochrane risk-of-bias tool for randomized trials (RoB2) [34]. The tool consists of 5 domains addressing different types of bias: randomization, deviations from the intended interventions, missing outcome data, measurement of the outcome and selection of the reported result. In any domain, appropriate questions had to be answered for each study. Next, the RoB2 algorithm is applied which evaluates the risks of the individual domains. Finally, an overall risk is calculated and expressed as “low” or “high” risk of bias, or the judgment can be expressed with “some concerns”.

Since blinding is very difficult in nutritional studies, it was also investigated which studies concealed ED manipulation from their participants to the highest possible extent. Secrecy was achieved by reducing visual, sensory and taste differences between the meals to a minimum.

## Results

### Study selection and categorization

**Fig. 1:**
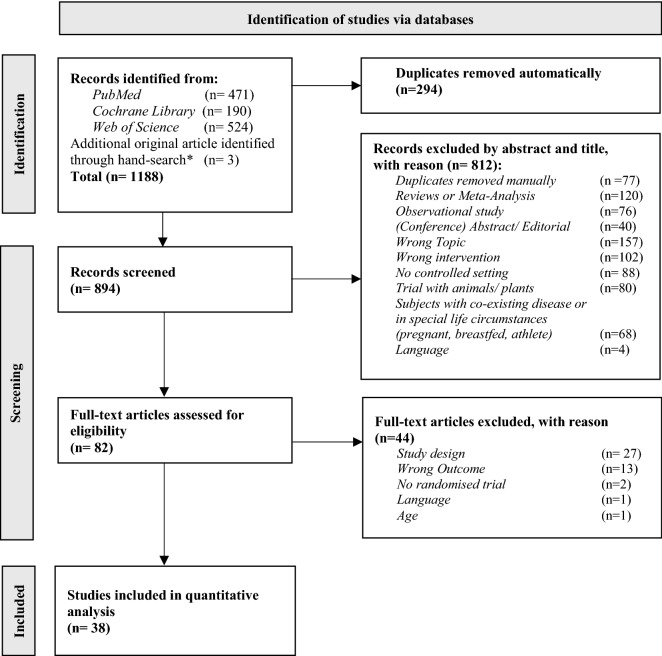
PRISMA flowchart for study inclusion. *Hand-search via database Ovid representative for Cochrane Library Search Strategy

The literature search process for identification of eligible studies is shown in Fig. 1. Out of 1188 identified studies, 38 RCTs remained for analysis.

### Summary of study characteristics

**Fig. 2:**
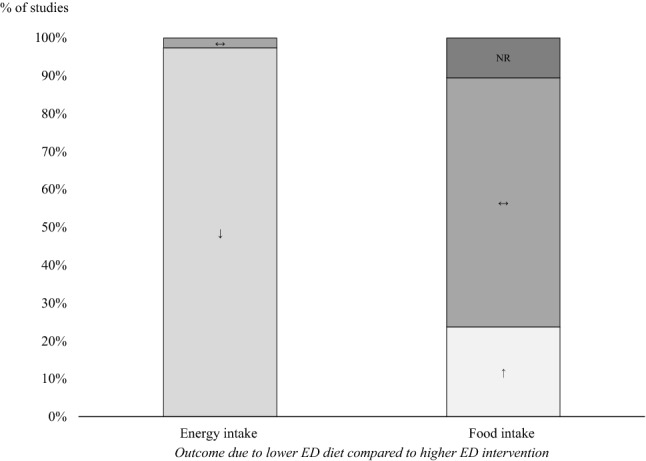
Changes in energy intake and food intake after lower energy density (ED) in comparison to higher ED diet across studies. Energy intake, food intake: ↑ intake is higher with lower ED than with higher ED intervention; ↓ intake is lower with lower ED than with higher ED intervention, ↔ no significant differences between lower ED and higher ED intervention; *NR* not reported

A detailed overview of the characteristics for the single trials is presented in Table 1. The characteristics across the studies are given in the text and summarized in Fig. 2.

**Table 1:** Summarized study characteristics for crossover trials

Author (year)	Country	Intake length (*i*), washout length (*w*)	Participants *N* (A); sex (f %); age [y] mean (SD)	Manipulated ED [kcal/g] of total meal (manipulated + if available unmanipulated items)	Consumed ED [kcal/g] of total meal (manipulated + if available unmanipulated items)	Outcomes
*Studies with entrée design (adults)*						
Bell and Rolls [46]	US	*i:* 6 × 1 meal	*N*: 46 (36); f: 100%;	LED: 1.1	LED: 1.1	EI [LED]: ↓
		*﻿w:* 5 x ≥ 5 days	age: S1: 26.9 (7.9), S2: 25.8 (1.3)	HED: 1.4	HED: 1.5	FI [LED]: ↔
Blatt et al. [47]	US	*i:* 3 × 1 day	*N*: 48 (41); f: 51.2%;	LED: 2.0 [+ side dishes; ED N.A.]	N.A.	EI [LED]: ↓
		*w:* 2 × 7 days	age: m: 24.4 (4.5), f: 23.9 (5.5)	HED: 2.6 [+ side dishes; ED N.A.]		FI [LED]: ↔
Cheskin et al. [49]	US	*i:* 2 × 4 meals	*N*: 76 (54); f: 66.7%; age: 35.5 (N.R.)	LED: 2.0	N.A.	EI [LED]: ↓
		*w:* 1 × 3 days		HED: 4.6		FI [LED]: ↔
Devitt and Mattes [50]	US	*i:* 4 days	*N*: 26 (20); f: 45.0%; age: 22.6 (5.8)	LED: 1.5	LED: 1.5	EI [LED]: ↓
		*w:* N.R.		HED: 2.6	HED: 2.6	FI [LED]: ↔ /↑
*Hogenkamp et al. [52]	NL	*i:* 4 × 5 meals	*N*: 118 (105); f: 56.2%; age: 22.0 (3.0)	LED: 0.5	LED: 0.5	EI [LED]: ↓
		*w:* 3 × 2 days		HED: 1.4	HED: 1.4	FI [LED]: ↑
Hogenkamp et al. [53]	NL	*i:* 4 × 4 days	*N*: 38 (27); f: 66.7%; age: 21.0 (2.4)	LED: 0.3	N.A.	EI [LED]: ↓
		*w:* 3 × 3 days		HED: 1.3		FI [LED]: ↑
Karl et al. [54]	US	*i:* 4 × 1 meal	*N*: 20 (20); f: 40.0%; age: 30.0 (11.0)	LED: 1.2	LED: 1.2	EI [LED]: ↓
		*w:* yes. but N.R.		HED: 1.6	HED: 1.6	FI [LED]: ↔
Kral et al. [55]	US	*i:* 3 × 1 day	*N*: 40 (40); f: 100%;	LED: 1.3 [+ side dishes; ED N.A.]	LED: 1.1	EI [LED]: ↓
		*w:* 2 × 6 days	age: S1: 20.5 (3.1), S2: 21.8 (2.7)	HED: 1.8 [+ side dishes; ED N.A.]	HED: 1.4	FI [LED]: ↑
Kral et al. [56]	US	*i:* 6 × 1 day	*N*: 45 (39); f: 100%; age: 23.4 (1.0)	LED: 1.3	LED: 1.1	EI [LED]: ↓
		*w:* 5 × 7 days		HED: 1.8	HED: 1.6	FI [LED]: ↑
McCrickerd et al. [57]	SGP	*i:* 4 × 1 meal	*N*: 61 (58); f: 53.5%;	LED: 0.6	N.A.	EI [LED]: ↓
		*w:* 3 x ≥ 3 days	age: m: 25.6 (5.3), f: 23.5 (3.5)	HED: 1.0		FI [LED]: ↔
Poppitt and Swann [58]	UK	*i:* 2 × 12 days	*N*: 6 (5); f: 0%; age: 35.0 (8.9)	LED: 0.9	N.A.	EI [LED]: ↓
		*w:* 1 × 3 days		HED: 2.0		FI [LED]: ↔
Rolls et al. [61]	US	*i:* 4 × 2 days	*N*: 25 (24); f: 100%; age: 21.9 (3.4)	LED: 1.6	LED: 1.6	EI [LED]: ↓
		*w:* 3 × 7 days		HED: 2.1	HED: 2.1	FI [LED]: ↔
Rolls et al. [62]	US	E1: *i:* 6 × 1 meal (*w:* 5 × 7 days)	*N*: 100 (97); f: 49.5%;	LED: 1.2	LED: 1.2	EI [LED]: ↓
			age E1:m: 26.8 (6.0), f: 26.7 (7.8);			
		E2: *i:* 6 × 1 meal (*w:* 5 × 7 days)	age E2:m: 24.8 (6.4), f: 28.5 (7.4)	HED: 1.3	HED: 1.3	FI [LED]: ↔
Silver et al. [63]	US	*i:* 6 meals/wk over 7 months	*N*: 52 (45); f: 68.9%; age: 84.4 (1.0)	LED: 1.1	N.A.	EI [LED]: ↓
		*w:* N.R.		HED: 2.2		FI [LED]: ↔
Stubbs, Harbron et al. [65]	UK	*i:* 3 × 9 days	*N*: 6 (6); f: 0%; age: 41.8 (10.6)	LED: 1.2	LED: 1.0	EI [LED]: ↓
		*w:* NR		HED: 1.7	HED: 1.5	FI [LED]: ↑
Stubbs, Ritz et al. [67]	UK	*i:* 3 × 16 days	*N*: 7 (7); f: 0%; age: 36.9 (7.6)	LED: 1.2	N.A.	EI [LED]: ↓
		*w:* N.R.		HED: 1.7		FI [LED]: ↔
Stubbs, Johnstone, Harbron et al. [64]	UK	*i:* 2 × 16 days	*N*: 6 (6); f: 0%; age: 32.2 (5.3)	LED: 0.9	LED: 0.8	EI [LED]: ↓
		*w:* 1 x ≥ 7 days		HED: 1.5	HED: 1.6	FI [LED]: ↑
Stubbs, Johnstone, O’Reilly et al. [66]	UK	*i:* 3 × 16 days	*N*: 6 (6); f: 0%; age: 30.0 (12.8)	LED: 0.9	LED: 0.9	EI [LED]: ↓
		*w:* 2 x ≥ 7 days		HED: 1.8	HED: 1.7	FI [LED]: ↑
Williams et al. [69]	US	*i:* 4 × 1 day	*N*: 62 (59); f: 49.2%;	LED: 1.4 [+ side dishes; ED N.A.]	LED: 1.5	EI [LED]: ↓
		*w:* 3 × 7 days	age: m: 26.1 (5.4), f: 25.6 (5.9)	HED: 1.8 [+ side dishes; ED N.A.]	HED: 1.7	FI [LED]: ↔
Williams et al. [68]	US	*i:* 6 × 1 meal	*N*: 53 (46); f: 100%; age: 25.4 (5.4)	LED: 1.3	LED: 1.3	EI [LED]: ↓
		w: 5 × 7 days		HED: 1.7	HED: 1.7	FI [LED]: ↔
Yeomans et al. [70]	UK	E1: *i:* 4 × 1 meal (*w:* 3×)	*N*: 16(16); f: 0%; age: 21.4 (1.6)	LED: 0.9	N.A.	EI [LED]: ↓
		E2: *i:* 8 × 1 meal (*w:* 7×)		HED: 2.0		FI [LED]: ↔ /↑
Yeomans et al. [71]	UK	*i:* 8 × 1 meal	*N*: 32 (32); f: 0%;	LED: 0.6	N.A.	EI [LED]: ↓
		*w:* 7×	age: S1: 22.3 (2.4), S2: 22.9 (3.8),	HED: 1.7		FI [LED]: ↔
			S3: 22.6 (2.8), S4: 22.5 (2.1)			
*Studies with entrée design (children)*						
Fisher et al. [35]	US	*i:* 4 × 1 meal	*N*: 53 (53); f: 52.8%; age: 5–6 (N.R.)	LED: 1.3 + 0.8	LED: 1.1	EI [LED]: ↓
		*w:* 3 × 7 days		HED: 1.8 + 0.8	HED: 1.3	FI [LED]: ↔
Johnson et al. [36]	US	*i:* 1–2 meals/ wk	*N*: N.R. (21); f: 47.6%;	LED_E1_: 1.1, HED_E1_: 2.2	LED_E1_: 1.1, HED_E1_: 2.2	EI [LED]: ↓
		*w:* N.R.	age: E1: 4.0 (0.7). E2: 2.8 (0.3)	LED_E2_: 1.1, HED_E2_: 2.3	LED_E2_: 1.1, HED_E2_: 2.3	FI [LED]: ↔
Kling, Roe, Keller et al. [37]	US	*i:* 6 × 1 meal	*N*: 131 (120); f: 49.2%; age: 4.4 (1.1)	LED: 0.8	LED: 0.8	EI [LED]: ↓
		w: 5 × 7 days		HED: 1.1	HED: 1.1	FI [LED]: ↔
Kling, Roe, Sanchez et al. [38]	US	*i:* 4 × 1 meal	*N*: 143 (125); f: 46.4%; age: 4.2 (1.1)	LED: 0.4 + 1.3	LED: 0.9	EI [LED]: ↔
		*w:* 3 × 7 days		HED: 0.6 + 1.3	HED: 1.0	FI [LED]: ↔
Leahy, Birch, Fisher et al. [42]	US	*i:* 4 × 1 meal	*N*: 75 (61); f: 50.8%;	LED: 0.8	LED: 0.7	EI [LED]: ↓
		*w:* 3 × 7 days	age: m: 4.5 (0.6). f: 4.3 (0.6)	HED: 0.9	HED: 0.9	FI [LED]: ↔
Leahy, Birch & Rolls [41]	US	*i:* 2 × 2 days	*N*: 29 (26); f: 61.5%; age: 4.2 (0.5)	LED: 0.9	LED: 1.0	EI [LED]: ↓
		*w:* 1 × 12 days		HED: 1.1	HED: 1.2	FI [LED]: ↔
Olsen et al. [43]	DK	*i:* 2 × 1 meal	*N*: N.R. (74); f: 60.8%; age: 5.6 (0.8)	LED: 0.9	LED: 0.9	EI [LED]: ↓
		*w:* N.R.		HED: 1.4	HED: 1.5	FI [LED]: ↔
Smethers et al. [44]	US	*i:* 3 × 5 days	*N*: 56 (49); f: 46.9%; age: 4.3 (0.7)	LED: 0.9	LED: 0.9	EI [LED]: ↓
		*w:* 2 × 7 days		HED: 1.0	HED: 1.1	FI [LED]: ↔
Spill et al. [45]	US	*i:* 3 × 1 day	*N*: 49 (39); f: 53.8%; age: 4.7 (0.6)	LED: 1.6	LED: 1.3	EI [LED]: ↓
		*w:* 2 × 6 days		HED: 2.0	HED: 1.5	FI [LED]: ↔
*Studies with preload design (adults)*						
Blatt et al. [48]	US	*i:* 4 × 1 day	*N*: 73 (68); f: 58.8%;	LED: 1.0 + 1.7	LED: 1.2	EI [LED]: ↓
		*w:* 3 × 7 days	age: m: 26.8 (5.8); f: 27.6 (7.0)	HED: 1.6 + 1.7	HED: 1.6	FI [LED]: ↑
Gray et al. [51]	UK	*i:* 5 × 1 meal	*N*: 18 (18); f: 0%; age: 26.0 (5.2)	LED: 0.3 + N.A.	N.A.	EI [LED]: ↓
		*w:* 4 × 2–14 days		HED: 1.0 + N.A.		FI [LED]: N.R.
*Hogenkamp et al. [52]	NL	*i:* 2 × 5 meals	*N*: 118 (105); f: 56.2%; age: 22.0 (3.0)	LED: 0.5 + 2.7	LED: 1.2	EI [LED]: ↓
		*w:* 1 × 28 days		HED: 1.4 + 2.7	HED: 1.8	FI [LED]: ↑
Rolls et al. [59]	US	*i:* 2 × 1 meals	*N*: 24 (24); f: 100%; age: 21.8 (4.4)	LED: 0.1 + 4.5	LED: 1.5	EI [LED]: ↓
		*w:* 1 × 7 days		HED: 0.5 + 4.5	HED: 1.6	FI [LED]: ↔
Rolls et al. [60]	US	*i:* 7 × 1 meal	*N*: 50 (42); f: 100%; age: 26.3 (7.8)	LED: 0.3 + 2.0	N.A.	EI [LED]: ↓
		*w:* 6 × 7 days		HED: 1.3 + 2.0		FI [LED]: ↔
Yeomans et al. [72]	UK	*i:* 6 × 1 meal	*N*: NR (36); f: 50.0%; age: 21.9 (3.2)	LED: 0.3 + N.A.	N.A.	EI [LED]: ↓
		*w:* 5 x		HED: 0.9 + N.A.		FI [LED]: N.R.
*Studies with preload design (children)*						
Kral et al. [39]	US	*i:* 3 × 1 meal	*N*: 94 (94); f: 53.2%; age: 8.9 (2.3)	LED: 0.6 + 0.6	N.A.	EI [LED]: ↓
		*w:* 2 × 7 days		HED: 1.0 + 0.6		FI [LED]: N.R.
Kral et al. [40]	US	*i:* 2 × 1 meal	*N*: 212 (212); f: 54.7%;	LED: 1.0 + 2.1	N.A.	EI [LED]: ↓
		*w:* 1 × 7 days	age: S1: 8.3 (0.7), S2/S3: 8.3 (0.8)	HED: 1.6 + 2.1		FI [LED]: N.R.

Consumed ED was calculated with energy intake (EI) and food intake (FI), from total meal (manipulated + unmanipulated food components); EI and FI after lower ED intervention compared to the higher ED diet: ↑: increase, ↓: decrease, ↔ : no difference, *: study with > 1 assignment

*Abbreviations: A* analyzed sample size, *DK* Denmark, *E* trial, *ED* energy density, *f* female, *HED* higher energy density condition, *i* intervention length, *LED* lower energy density condition, *m* male, *N* number of participants, *N.A.* not available/not calculable, *NL* Netherlands, *N.R.* not reported, *S* subgroup, *SD* standard deviation, *SGP* Singapore, *UK* United Kingdom, *US* United States of America, *w* washout period, *wk* week, *y* years

Among the 38 trials, most were conducted in America (*n* = 25; 65.8%), followed by Europe (*n* = 12; 31.6%) and Asia (*n* = 1; 2.6%). These were RCTs published between 1988 and 2020.

Total participants from the eligible trials for quantitative analysis of RCTs were 1831 participants; of which 874 were children or adolescents, whereas 957 were adults.

For the children studies (*n* = 11, [35–45]), the median age was 4.6 [4.3–8.3] years, covering the ages between 2 and 12 years. Girls represented 53% of the participants. The median BMI percentile was 59 [56.1–68.8], with a range of 42.5–94.5. The majority of the trials investigated the effects on children of normal weight and only two trials included children with overweight in their research. Two studies offered the children a manipulated preload and analyzed the subsequent *ad libitum* meal, whereas nine studies provided children with a manipulated entrée. All of the preload studies manipulated only one meal per day, in contrast to 33% (*n* = 3) of entrée studies lasting longer than one meal.

In the studies with focus on adults (*n* = 27, [46–72]), the median age was 25 [22.5–26.9] years, covering the ages between 21 and 84 years. Women represented 52% of the participants. The general median BMI for adults was 23.2 [22.4–24.0] kg/m^2^, with a range of 21–34.7 kg/m^2^. One study examined the effects of ED manipulation on adults with overweight, whereas the remaining studies focused on the effects on participants of normal weight. Five of the 27 studies offered a preload and analysed the subsequent *ad libitum* meal, whereas 78% of the studies (*n* = 21) served a manipulated entrée. One study applied both preload and entrée design [52].

As a result, a total of eight studies [39, 40, 48, 51, 52, 59, 60, 72] served a compulsory manipulated preload and measured the food intake of the following unmanipulated *ad libitum* test meal. A total of 31 studies modified an entrée and measured the *ad libitum* intake of this entrée. The length of all interventions ranged from 2 to 48 days with a median length of 6 [4–8] days. A washout period was performed between the dietary interventions in each of the 38 crossover studies. The washout periods lasted minimum 2 and maximum 28 days with a median length of 6 [6–7] days. When the effects of the ED manipulation were investigated for only a single and not multiple meals (*n* = 15), lunch was mostly used as the intervention meal (*n* = 14), followed by breakfast (*n* = 8) and dinner (*n* = 1). There were test foods with a median ED of 1.1 kcal/g [0.80–1.2], ranging from 0.1 to 2.0 kcal/g in the lower ED intervention and a median ED of 1.5 kcal/g [1.10–1.80] with a range of 0.5–4.6 kcal/g in the higher ED intervention. The ED consumed correlates linearly with the ED served (*R*^2^_lowerED_ = 0.9181, *R*^2^_higherED_ = 0.9494). In 22 studies, in addition to ED manipulation, portion size (*n* = 12), sensory quality (e.g. viscosity, taste, color or palatability, (*n* = 7)), other macronutrient compositions (*n* = 2) or information regarding a manipulation (*n* = 1) were varied. This resulted in 2 × 2 or 3 × 2 factorial crossover designs, with the ED manipulation supplemented by one or two of the aforementioned manipulations in each case. In most trials, energy intake was the primary endpoint, only 2 studies (5%) considered energy intake as secondary endpoint.

### Summary of study outcomes

Overall, the heterogeneity of studies was high with respect to study design, sample size and research question.

#### Energy intake

Energy intake was compared between the lower ED and higher ED interventions at qualitative and quantitative levels for all 38 studies. The results of the qualitative analysis are presented as an overview in Table 1 and across studies in Fig. 2.

Thirty-seven studies (97%) indicated that energy intake was lower with lower ED than with higher ED intervention. Only one study [38] showed no change in energy intake in children after the lower ED intervention, indicating that the same amount of energy was consumed via both the lower ED and higher ED diets. There were also no differences between participants with normal-weight or overweight/obesity.

**Fig. 3:**
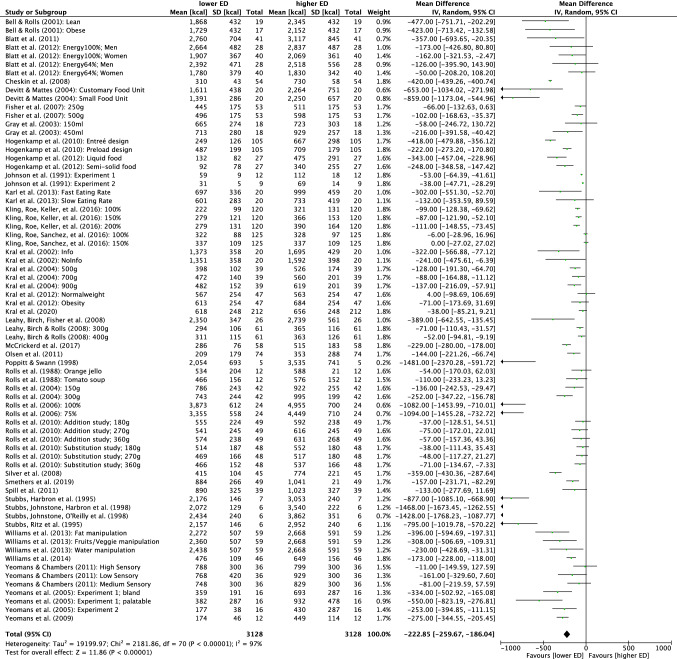
Quantitative analysis of energy intake of all randomized controlled trials receiving either lower energy density (ED) or higher ED meals. The forest plot displays effect estimates and 95% confidence intervals (CI) for individual studies and the summary of findings. Additionally, for each study mean energy intake [kcal], standard deviation (SD) [kcal] and the number of total participants of both lower ED and higher ED conditions are presented. *IV* inverse-variance

For quantitative analysis, the 38 multifactorial crossover studies were split according to their study conditions [32] resulting in 71 effects. The result of the quantitative analysis is presented as a forest plot in Fig. 3. Energy intake was reduced in the lower ED relative to higher ED conditions (mean energy intake difference – 223 kcal (95% CI: – 259.7, – 186.0); *p* < 0.001). However, the heterogeneity was high with *I*^2^ = 97% despite the applied random effect model.

To investigate the sources of heterogeneity, subgroup analyses were performed according to participants’ age (subgroup analysis 1), meal type (subgroup analysis 2) and intervention length per day (subgroup analysis 3).

##### Subgroup analysis 1: effects of participants’ age (children versus adults) on energy intake

This analysis aimed at reducing heterogeneity by dividing the studies according to the age of the participants (Figure S1). Although heterogeneity of adult studies remained high (*I*^2^ = 94%), was still reduced in the lower ED relative to higher ED interventions (mean energy intake difference – 302 kcal (95% CI: – 358.9, – 246.4); *p* < 0.001). In contrast, for trials with children heterogeneity was reduced (*I*^2^ = 80%) and accompanied by a drop in efficacy (mean energy intake difference – 65 kcal (95% CI: – 83.5, – 47.0); *p* < 0.001).

##### Subgroup analysis 2: effects of meal type (preload versus entrée design) on energy intake

In the analysis examining preload versus entrée studies (Figure S2), lower ED conditions were associated with a reduction in energy intake (mean energy intake difference – 111 kcal (95% CI: – 159.2, – 62.5); *p* < 0.001) and manipulated entrées (mean energy intake difference – 261 kcal (95% CI: – 304.6, – 217.9); *p* < 0.001), although treatment effects were significantly greater for manipulated entrées than manipulated preloads (*p* < 0.001). Heterogeneity decreased when analyzing preload studies (*I*^2^ = 66%), but remained high for entrée studies (*I*^2^ = 98%).

##### Subgroup analysis 3: effects of intervention length (1 meal versus > 1 meal) on energy intake

Lastly, this analysis distinguished according to length of intervention period (Figure S3). Effectiveness of the multiple meal interventions was superior to single meal interventions (*p* < 0.001), indicating a persistence effect of the ED manipulation. However, heterogeneity remained high for both single interventions (*I*^2^ = 97%) and multiple interventions (*I*^2^ = 92%).

##### Combination of subgroup analysis 1 + 2: effects of age and meal type on energy intake

**Fig. 4:**
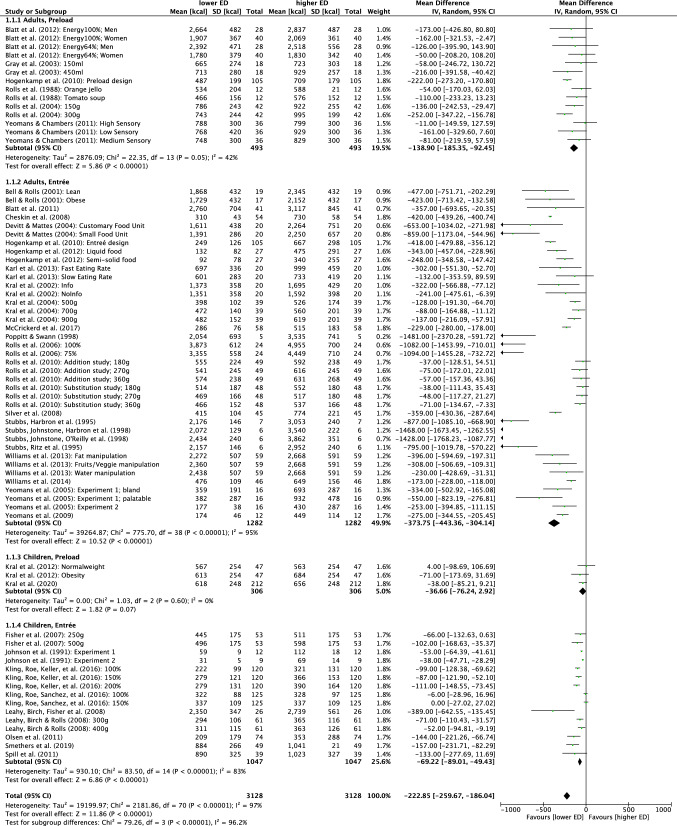
Quantitative analysis of age (children versus adults) and meal type (preload versus entrée) on energy intake of randomized controlled crossover trials in humans receiving either lower energy density (ED) or higher ED diets. The forest plot displays effect estimates and 95% confidence intervals (CI) for individual studies and the summary of findings. Additionally, for each study mean energy intake [kcal], standard deviation (SD) [kcal] and the number of total participants of both lower ED and higher ED conditions are presented. *IV* inverse-variance

Heterogeneity decreased when subgroups 1 and 2 were combined for analysis (Fig. 4). Here, heterogeneity decreased slightly when analyzing only children/entrée studies (*I*^2^ = 83%), but dropped strongly for children/preload interventions (*I*^2^ = 0%). Nevertheless, no significant subgroup effect for preloads was found (*p* = 0.07). A reduced heterogeneity was found when analyzing adult/preload studies (*I*^2^ = 42%) but adult/entrée studies remained high in their heterogeneity (*I*^2^ = 95%). Subgroup differences were significant (*p* < 0.001). Energy intake was lower in all subgroups in the lower ED relative to the higher ED intervention with mean energy intake differences of – 69, – 37, – 374 and – 139 kcal for children/entrée, children/preload, adults/entrée, and adults/preload studies, respectively. No further subgroup analysis could reduce heterogeneity, which is why no further analyses are mentioned.

Overall, the subgroup analyses were able to explain the heterogeneity of studies in the full analyses. The data of the RCTs clearly demonstrated that the lower ED intervention reduced the energy intake compared to the higher ED intervention.

#### Food intake 

To improve understanding of the findings regarding the outcome of energy intake in lower ED versus higher ED interventions, the amount of food intake in both conditions is presented in the following. The results regarding food intake for the single studies are presented at qualitative level for 38 studies as an overview in Table 1 and across studies in Fig. 2.

Twenty-five studies showed no significant difference in food intake between the two interventions (66%), nine studies reported an increase in food intake after a lower ED meal in comparison to a higher ED meal (24%) and the remaining studies (*n* = 4, 10%) did not report on food intake. Except for one study [28], all of the studies with higher food intake in the lower ED than in the higher ED diet were studies in which adult study participants received a manipulated entrée.

**Fig. 5:**
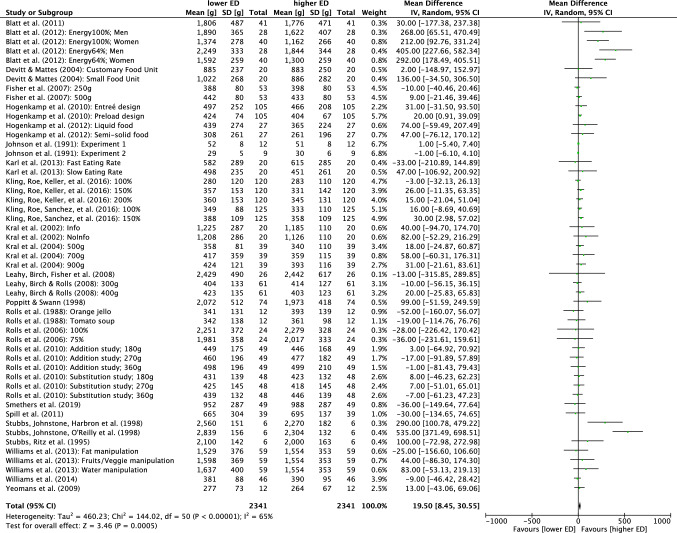
Quantitative analysis of food intake of randomized controlled trials receiving either lower energy density (ED) or higher ED meals. The forest plot displays effect estimates and 95% confidence intervals (CI) for individual studies and the summary of findings. Additionally, for each study mean energy intake [kcal], standard deviation (SD) [kcal] and the number of total participants of both lower ED and higher ED conditions are presented. *IV* inverse-variance

For quantitative analysis, 26 studies were included and the results are presented as a forest plot in Fig. 5. Independent of ED manipulation, the amount of food consumed was rather similar between the intervention groups, although in some cases food intake was slightly increased in the lower ED test meals (mean food intake difference 20 g (95% CI: 8.5, 30.6); *p* < 0.001), meaning marginally more food was eaten in lower ED interventions. The heterogeneity from the trials (*I*^2^ = 65%) required no further exploration.

### Relationship between delta ED consumed and delta energy intake

**Fig. 6:**
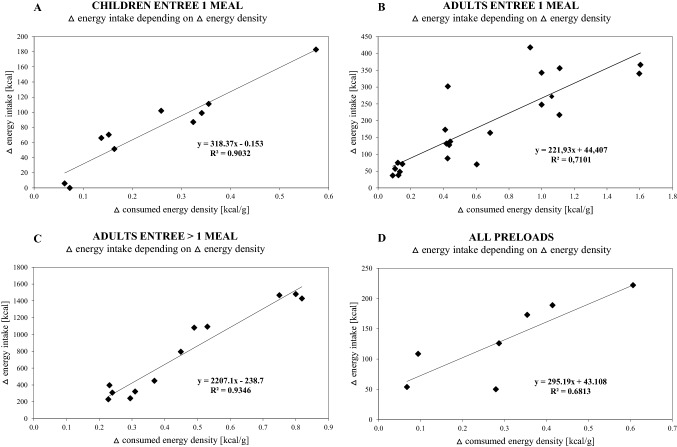
Relationship between △ energy density (ED) and △ energy intake. Data of △ ED (lower versus higher ED condition of each single study; △ kcal/g) with the corresponding △ energy intake (lower versus higher ED condition of each single study, kcal) are displayed. *A:* Entrée studies in children, 1 meal interventions. *B:* Entrée studies in adults, 1 meal interventions. *C:* Entrée studies in adults, > 1 meal interventions. *D:* Preload studies in children and adults

Substantial linear relationships between △ consumed ED (lower versus higher ED condition) and △ energy intake were found across different meal types and age (Fig. 6). The linear relationship in children with entrée design and 1 meal per day (A) was stronger (*R*^2^ = 0.90) than in adults (*R*^2^ = 0.71, B). In adults, the linear relationship became very strong when analyzing the entrée design including more than 1 intervention meal per day (*R*^2^ = 0.93; C). Studies with preload design (D) also showed a clear linear relationship (*R*^2^ = 0.68; separation between adults and children was not possible due to the small sample size).

### Risk of bias

**Fig. 7:**
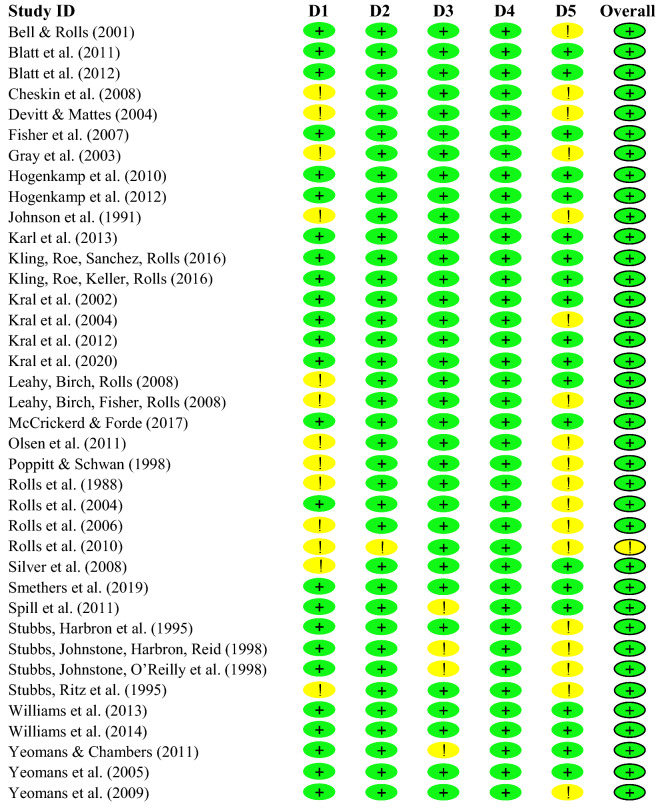
Risk of bias. *D1* Randomization process, *D2* Deviations from the intended interventions, *D3* Missing outcome data, *D4* Measurement of the outcome, *D5* Selection of the reported results. + : Low risk of bias, ! : Some concerns in risk of bias, − : High risk of bias

Figure 7 is a risk of bias summary showing the review authors judgements about each risk of bias item for each included study. The overall risk of bias was low in 37 studies and with some concerns in 1 study. None of the studies were identified with a high risk. All of the 38 trials were analyzed per protocol rather than intention-to-treat.

Only in 4 out of 38 studies (11%) the ED condition was evident to the participant. All other studies tried to conceal the ED condition to the highest possible extent. However, it is unclear if this goal was achieved in the single studies. Excluding the 4 studies (*n* = 430, [49, 55, 62, 63]) with overt manipulation did not influence the findings and no sub-group differences were observed between overt and covert manipulation (data not shown). Hence, all studies were included in this meta-analysis.

## Discussion

This systematic review and meta-analysis summarized the data of RCT studies investigating the influence of dietary ED on energy and food intake. Both hypotheses were qualitatively and quantitatively confirmed, showing that the lower ED reduced energy intake compared to the higher ED intervention, while food intake was unaffected. Meta-analysis data clearly indicated decreased energy intake with lower ED interventions than with higher ED interventions, as supported by another meta-analysis that included both RCTs and non-randomized controlled trials [19]. Moreover, a clear linear relationship was demonstrated between delta ED and delta energy intake, resulting in a lower energy intake with lower ED food. In contrast, food intake exhibits a non-linear relationship in regard to portion size [73] and rather a person’s consumption approaches an asymptote after exceeding a certain portion size [74, 75]. In comparison to the meta-analysis by Robinson et al*.* [19], our impacts were less strong but still substantial. Reasons for this may be due to different approaches taken, as the presented analysis only incorporated RCTs, where each participant always served as their own control and food intake had to be measured objectively by the investigators. Moreover, this review also comprised studies including ≥ 1 manipulated meal, resulting in other foci of analysis (meal type, 1 meal versus > 1 meal).

Subgroup analyzes were performed to produce the most homogeneous results possible, with highest homogeneity of the data found by combining the subgroups age (children versus adults) and meal type (preload versus entrée). The division of the subgroups ‘preload’ versus ‘entrée’ appeared essential due to their differing mechanism (inter-meal-satiety in preload versus intra-meal-satiation in *ad libitum* entrées) [76]. Preload studies with low ED foods such as salad [60], fruit [77] or soup [78] reported a reduction in energy intake in the following *ad libitum* meal. Nevertheless, the participants showed a non-significant trend of compensation for the reduction in energy intake [23]. In line, our data suggests that some kind of compensation must have taken place for the ED manipulation in preload studies, because the differences in energy intake between lower versus higher ED conditions were smaller in preload (– 118 kcal) than in *ad libitum* entrée studies (– 261 kcal). However, it should be noted that the differences between lower and higher ED conditions were generally smaller in preload than in *ad libitum* entrée studies. In regards to the entrée design, we observed a strong linear relationship between delta ED and delta energy intake in children and adults, indicating a lack of compensation in energy intake in this study design.

As hypothesized, food intake remained constant which removes the possibility of a compensation via the amount of food. As reported earlier [79, 80], satiety/satiation processes are not only dependent on the caloric content of the food but also on gastric capacity. Studies in which subjects received intragastric infused preloads bypassing sensory stimuli showed that the volume was more decisive for the feeling of satiety than the energy content [80, 81]. It has also been shown that the decreasing hedonic response to foods during consumption depends more on the volume of the food than on its energy content [82]. In addition, the duration of oral exposure is at least as important for the reduction of energy intake as the gastric filling volume [83, 84]. Similarly, it is possible that individuals use prior learning experiences to consume similar portion sizes under controlled conditions, regardless of ED [2]. In total, a complex interplay of cognitive, sensory, neural, gastrointestinal and hormonal influences [11] is involved and the above-described mechanisms should be seen in the context of the complex regulation of hunger and satiety. The topic has already been excellently reviewed elsewhere [76, 85].

Overall, the results of this conservative meta-analysis are substantial and, together with the findings of the linear relationship between ED and caloric intake, of high relevance for body weight management. In practice, this means that reducing the ED of food allows individuals to eat satiating quantities while at the same time consuming less energy. Additionally, ED lowering strategies are flexible and diverse and can be adapted to different dietary patterns, food preferences and cultural characteristics [2, 17, 18]. In line, two randomized controlled trials have shown that already primary school children can understand and are able to apply ED knowledge to their daily routine, even at six months follow-up [86, 87]. Taken together, these are strong arguments for increase integration of ED manipulation as dietary strategy in prevention and clinical practice.

### Strengths and weaknesses of the systematic review and meta-analysis

Overall, this systematic review and meta-analysis has several limitations and strengths. A clear strength is the methodological approach taken according to PRISMA [26] and Cochrane criteria [32]. To provide homogeneity of the trials, the search was limited to RCTs and therefore, the probability of comparability between groups and the validity of the results was very high. In each study, participants served as their own control of the intervention regarding the ED manipulation, which further increased comparability. A limiting factor is the possibility for performance bias, as a common problem with nutritional studies in a controlled environment is that blinding of the participants and research personnel is impossible [88]. Nevertheless, most of the trials were covertly performed and a part of the meal was substituted with a similar lower ED alternative. Studies indicate that a covert incorporation of puréed vegetables into entrées significantly reduced energy intake in children [45] and adults [47]. Since only RCT studies were summarized and participants blinded to the best of the ability, it can be expected that the results are due to the nature of the ED manipulation. Despite clear eligibility criteria, the heterogeneity of the studies was high at the descriptive and meta-analytical levels, and therefore, subgroup analyses were performed, reducing heterogeneity to some degree. Finally, this meta-analysis had not yet been conducted in a comparable setting, meaning that it was unprecedented and provided new results, especially in the context of the impact of ED manipulation in different age groups and different manipulated meal types. By using exclusion criteria and introducing a moderator variable to code the studies according to their methodological quality, very strong methodological studies without a risk of bias were included. The subgrouping of consistent dependent variables enabled an interpretation of the resulting mean effect size. The clear tendency of all hypotheses was confirmed with a sufficient degree of certainty, which is why the importance of this systematic review and meta-analysis is very high and of interest for future preventive and therapeutic approaches.

## Conclusion

In conclusion, energy intake in humans, irrespective of age, meal type and intervention length is determined by the ED of a meal. The magnitude of the effect is substantial and the relationship between consumed ED and energy intake linear. Thus, manipulating the ED of foods has the potential to be a powerful tool for body weight management in prevention and therapy.

## Supplementary Information

Below is the link to the electronic supplementary material.

Supplementary file1 (PDF 4332 KB)

## Data Availability

Not applicable.
