# Supplementary material for: Impact of energy density on energy intake in children and adults: a systematic review and meta-analysis of randomized controlled trials
Source: Eur J Nutr. 2022 Dec 2;62(3):1059–76. doi: 10.1007/s00394-022-03054-z (PMC10030411; doi:10.1007/s00394-022-03054-z)

Systematic Review & Meta-Analysis

Impact of Energy Density on Energy Intake in Children and Adults – a Systematic Review and Meta-Analysis of Randomized Controlled Trials Supplementary Information

European Journal of Nutrition

Bea Klos[1], Jessica Cook[1], Letizia Crepaz[1], Alisa Weiland[1], Stephan Zipfel[1], Isabelle Mack[1*]

[1]University Hospital Tübingen, Department of Psychosomatic Medicine and Psychotherapy, Tübingen, Germany

*Correspondence: Dr. Isabelle Mack, University Hospital Tübingen, Internal Medicine VI, Department of Psychosomatic Medicine and Psychotherapy, Osianderstr. 5, 72076 Tübingen, Phone: +49-7071-2985614, Fax: +49-7071-294382, E-Mail: isabelle.mack@uni-tuebingen.de

Text S1 – Search strategy

**Database**	**Search term**
**Pubmed**	((((Manipulat*[tiab] OR Varying*[tiab] OR Variation*[tiab] OR Vary[tiab] OR Varied[tiab] OR Chang*[tiab] OR Alteration*[tiab] OR Alterning*[tiab]) AND "Energy Density"[tiab]) AND (Compar*[tiab] OR Group[tiab] OR control*[tiab] OR increase[tiab] OR decrease[tiab])) AND (("Energy Intake"[Mesh] OR Energy Intake[tiab] OR Caloric Intake[tiab] OR "Eating/physiology"[Mesh] OR Food Intake[tiab] OR macronutrient composition*[tiab] OR food composition*[tiab] OR nutrient composition*[tiab] OR laborator*[tiab] OR measure*[tiab] OR food setting*[tiab]) NOT ("Food Frequency Questionaire*"[tiab] OR FFQ[tiab]))) NOT (("Animals"[Mesh] NOT ("Humans"[Mesh] OR Human*[tiab])))
**Web of Science**	#1 ((Manipulat* OR Varying OR Variation* OR Vary OR Varied OR Chang* OR Alteration* OR Alterning*) AND (Energy NEXT Density)):ti,ab,kw<br>#2 (Compar* OR Group OR control* OR increase OR decrease):ti,ab,kw<br>#3 ([mh "Energy Intake"] OR ((Energy OR Caloric OR Food) NEXT Intake):ti,ab,kw OR (((macronutrient OR food OR nutrient) NEAR/3 composition*) OR laborator* OR measure* OR (food NEXT setting*)):ti,ab,kw) NOT (((Food NEXT Frequency NEXT Questionaire*) OR FFQ):ti,ab,kw)<br>#4 [mh "Animals"] NOT ([mh "Humans"] OR Human*:ti,ab,kw)

	#5	#1 AND #2 AND #3
	#6	#5 NOT #4
**Cochrane Library**	#1	TS=((Manipulat* OR Varying OR Variation* OR Vary OR Varied OR Chang* OR Alteration* OR Alterning*) AND "Energy Density" AND (Food OR Eating OR Nutrition OR Meal*))
	#2	TS=(Compar* OR Group OR control* OR increase OR decrease)
	#3	TS=((((Energy OR Caloric OR Food) NEAR/0 Intake) OR ((macronutrient OR food OR nutrient) NEAR/3 composition*) OR laborator* OR measure* OR "food setting*") NOT ("Food Frequency Questionaire*" OR FFQ))
	#4	TS=(Animal* NOT (Human* OR Patient* OR Women OR Woman OR Man OR Men OR Child OR Children))
	#5	#1 AND #2 AND #3
	#6	#5 NOT #4
**Ovid**	1	((Manipulat* or Varying or Variation$ or Vary or Varied or Chang* or Alteration$ or Alterning*).ti,ab.) and (Energy.ti,ab. adj Density.ti,ab.)
	2	(Compar* OR Group OR control* OR increase OR decrease).ti,ab.
	3	(exp Energy Intake/ or exp Eating/ph or ((Energy or Caloric or Food).ti,ab. adj Intake.ti,ab.) or ((macronutrient or food or nutrient).ti,ab. adj3 composition$.ti,ab.) or (laborator* or measure*).ti,ab. or (food.ti,ab. adj setting$.ti,ab)) not ("Food Frequency Questionaire$".ab,ti. or FFQ.ab,ti.)
	4	(exp Animals/ or animal$.ti,ab.) not (exp Humans/ or Human$.ti,ab.)
	5	1 and 2 and 3
	6	5 not 4

Figure S1 Quantitative analysis of the effects of age (adults versus children) on energy intake of randomized controlled crossover trials in humans receiving either lower energy density (ED) or higher ED diets

Study or Subgroup	lower ED Mean [kcal]	lower ED SD [kcal]	lower ED Total	higher ED Mean [kcal]	higher ED SD [kcal]	higher ED Total	Weight	Mean Difference IV, Random, 95% CI
**1.1.1 Adults**								
Bell & Rolls (2001): Lean	1,868	432	19	2,345	432	19	0.9%	−477.00 [−751.71, −202.29]
Bell & Rolls (2001): Obese	1,729	432	17	2,152	432	17	0.9%	−423.00 [−713.42, −132.58]
Blatt et al. (2011)	2,760	704	41	3,117	845	41	0.7%	−357.00 [−693.65, −20.35]
Blatt et al. (2012): Energy100%; Men	2,664	482	28	2,837	487	28	1.0%	−173.00 [−426.80, 80.80]
Blatt et al. (2012): Energy100%; Women	1,907	367	40	2,069	361	40	1.4%	−162.00 [−321.53, −2.47]
Blatt et al. (2012): Energy64%; Men	2,392	471	28	2,518	556	28	0.9%	−126.00 [−395.90, 143.90]
Blatt et al. (2012): Energy64%; Women	1,780	379	40	1,830	342	40	1.4%	−50.00 [−208.20, 108.20]
Cheskin et al. (2008)	310	43	54	730	58	54	1.8%	−420.00 [−439.26, −400.74]
Devitt & Mattes (2004): Customary Food Unit	1,611	438	20	2,264	751	20	0.6%	−653.00 [−1034.02, −271.98]
Devitt & Mattes (2004): Small Food Unit	1,391	286	20	2,250	657	20	0.8%	−859.00 [−1173.04, −544.96]
Gray et al. (2003): 150ml	665	274	18	723	303	18	1.2%	−58.00 [−246.72, 130.72]
Gray et al. (2003): 450ml	713	280	18	929	257	18	1.3%	−216.00 [−391.58, −40.42]
Hogenkamp et al. (2010): Entreé design	249	126	105	667	298	105	1.7%	−418.00 [−479.88, −356.12]
Hogenkamp et al. (2010): Preload design	487	199	105	709	179	105	1.8%	−222.00 [−273.20, −170.80]
Hogenkamp et al. (2012): Liquid food	132	82	27	475	291	27	1.6%	−343.00 [−457.04, −228.96]
Hogenkamp et al. (2012): Semi-solid food	92	78	27	340	255	27	1.6%	−248.00 [−348.58, −147.42]
Karl et al. (2013): Fast Eating Rate	697	336	20	999	459	20	1.0%	−302.00 [−551.30, −52.70]
Karl et al. (2013): Slow Eating Rate	601	283	20	733	419	20	1.1%	−132.00 [−353.59, 89.59]
Kral et al. (2002): Info	1,373	358	20	1,695	429	20	1.0%	−322.00 [−566.88, −77.12]
Kral et al. (2002): NoInfo	1,351	358	20	1,592	398	20	1.1%	−241.00 [−475.61, −6.39]
Kral et al. (2004): 500g	398	102	39	526	174	39	1.7%	−128.00 [−191.30, −64.70]
Kral et al. (2004): 700g	472	140	39	560	201	39	1.7%	−88.00 [−164.88, −11.12]
Kral et al. (2004): 900g	482	152	39	619	201	39	1.7%	−137.00 [−216.09, −57.91]
McCrickerd et al. (2017)	286	76	58	515	183	58	1.8%	−229.00 [−280.00, −178.00]
Poppitt & Swann (1998)	2,054	693	5	3,535	741	5	0.2%	−1481.00 [−2370.28, −591.72]
Rolls et al. (1988): Orange jello	534	204	12	588	21	12	1.6%	−54.00 [−170.03, 62.03]
Rolls et al. (1988): Tomato soup	466	156	12	576	152	12	1.5%	−110.00 [−233.23, 13.23]
Rolls et al. (2004): 150g	786	243	42	922	255	42	1.6%	−136.00 [−242.53, −29.47]
Rolls et al. (2004): 300g	743	244	42	995	199	42	1.6%	−252.00 [−347.22, −156.78]
Rolls et al. (2006): 100%	3,873	612	24	4,955	700	24	0.6%	−1082.00 [−1453.99, −710.01]
Rolls et al. (2006): 75%	3,355	558	24	4,449	710	24	0.7%	−1094.00 [−1455.28, −732.72]
Rolls et al. (2010): Addition study; 180g	555	224	49	592	238	49	1.7%	−37.00 [−128.51, 54.51]
Rolls et al. (2010): Addition study; 270g	541	245	49	616	245	49	1.6%	−75.00 [−172.01, 22.01]
Rolls et al. (2010): Addition study; 360g	574	238	49	631	268	49	1.6%	−57.00 [−157.36, 43.36]
Rolls et al. (2010): Substitution study; 180g	514	187	48	552	180	48	1.7%	−38.00 [−111.43, 35.43]
Rolls et al. (2010): Substitution study; 270g	469	166	48	517	180	48	1.7%	−48.00 [−117.27, 21.27]
Rolls et al. (2010): Substitution study; 360g	466	152	48	537	166	48	1.7%	−71.00 [−134.67, −7.33]
Silver et al. (2008)	415	104	45	774	221	45	1.7%	−359.00 [−430.36, −287.64]
Stubbs, Harbron et al. (1995)	2,176	146	7	3,053	240	7	1.2%	−877.00 [−1085.10, −668.90]
Stubbs, Johnstone, Harbron et al. (1998)	2,072	129	6	3,540	222	6	1.2%	−1468.00 [−1673.45, −1262.55]
Stubbs, Johnstone, O'Reilly et al. (1998)	2,434	240	6	3,862	351	6	0.7%	−1428.00 [−1768.23, −1087.77]
Stubbs, Ritz et al. (1995)	2,157	146	6	2,952	240	6	1.1%	−795.00 [−1019.78, −570.22]
Williams et al. (2013): Fat manipulation	2,272	507	59	2,668	591	59	1.2%	−396.00 [−594.69, −197.31]
Williams et al. (2013): Fruits/Veggie manipulation	2,360	507	59	2,668	591	59	1.2%	−308.00 [−506.69, −109.31]
Williams et al. (2013): Water manipulation	2,438	507	59	2,668	591	59	1.2%	−230.00 [−428.69, −31.31]
Williams et al. (2014)	476	109	46	649	156	46	1.8%	−173.00 [−228.00, −118.00]
Yeomans & Chambers (2011): High Sensory	788	300	36	799	300	36	1.5%	−11.00 [−149.59, 127.59]
Yeomans & Chambers (2011): Low Sensory	768	420	36	929	300	36	1.3%	−161.00 [−329.60, 7.60]
Yeomans & Chambers (2011): Medium Sensory	748	300	36	829	300	36	1.5%	−81.00 [−219.59, 57.59]
Yeomans et al. (2005): Experiment 1; bland	359	191	16	693	287	16	1.3%	−334.00 [−502.92, −165.08]
Yeomans et al. (2005): Experiment 1; palatable	382	287	16	932	478	16	0.9%	−550.00 [−823.19, −276.81]
Yeomans et al. (2005): Experiment 2	177	38	16	430	287	16	1.4%	−253.00 [−394.85, −111.15]
Yeomans et al. (2009)	174	46	12	449	114	12	1.7%	−275.00 [−344.55, −205.45]
**Subtotal (95% CI)**			**1775**			**1775**	**69.4%**	**−302.62 [−358.87, −246.36]**
Heterogeneity: Tau² = 34895.51; Chi² = 866.69, df = 52 (P < 0.00001); I² = 94%								
Test for overall effect: Z = 10.54 (P < 0.00001)								
**1.1.2 Children**								
Fisher et al. (2007): 250g	445	175	53	511	175	53	1.7%	−66.00 [−132.63, 0.63]
Fisher et al. (2007): 500g	496	175	53	598	175	53	1.7%	−102.00 [−168.63, −35.37]
Johnson et al. (1991): Experiment 1	59	9	12	112	18	12	1.8%	−53.00 [−64.39, −41.61]
Johnson et al. (1991): Experiment 2	31	5	9	69	14	9	1.8%	−38.00 [−47.71, −28.29]
Kling, Roe, Keller, et al. (2016): 100%	222	99	120	321	131	120	1.8%	−99.00 [−128.38, −69.62]
Kling, Roe, Keller, et al. (2016): 150%	279	121	120	366	153	120	1.8%	−87.00 [−121.90, −52.10]
Kling, Roe, Keller, et al. (2016): 200%	279	131	120	390	164	120	1.8%	−111.00 [−148.55, −73.45]
Kling, Roe, Sanchez, et al. (2016): 100%	322	88	125	328	97	125	1.8%	−6.00 [−28.96, 16.96]
Kling, Roe, Sanchez, et al. (2016): 150%	337	109	125	337	109	125	1.8%	0.00 [−27.02, 27.02]
Kral et al. (2012): Normalweight	567	254	47	563	254	47	1.6%	4.00 [−98.69, 106.69]
Kral et al. (2012): Obesity	613	254	47	684	254	47	1.6%	−71.00 [−173.69, 31.69]
Kral et al. (2020)	618	248	212	656	248	212	1.8%	−38.00 [−85.21, 9.21]
Leahy, Birch, Fisher et al. (2008)	2,350	347	26	2,739	561	26	1.0%	−389.00 [−642.55, −135.45]
Leahy, Birch & Rolls (2008): 300g	294	106	61	365	116	61	1.8%	−71.00 [−110.43, −31.57]
Leahy, Birch & Rolls (2008): 400g	311	115	61	363	126	61	1.8%	−52.00 [−94.81, −9.19]
Olsen et al. (2011)	209	179	74	353	288	74	1.7%	−144.00 [−221.26, −66.74]
Smethers et al. (2019)	884	266	49	1,041	21	49	1.7%	−157.00 [−231.71, −82.29]
Spill et al. (2011)	890	325	39	1,023	327	39	1.4%	−133.00 [−277.69, 11.69]
**Subtotal (95% CI)**			**1353**			**1353**	**30.6%**	**−65.24 [−83.50, −46.97]**
Heterogeneity: Tau² = 872.39; Chi² = 84.84, df = 17 (P < 0.00001); I² = 80%								
Test for overall effect: Z = 7.00 (P < 0.00001)								
**Total (95% CI)**			**3128**			**3128**	**100.0%**	**−222.85 [−259.67, −186.04]**
Heterogeneity: Tau² = 19199.97; Chi² = 2181.86, df = 70 (P < 0.00001); I² = 97%								
Test for overall effect: Z = 11.86 (P < 0.00001)								
Test for subgroup differences: Chi² = 61.88, df = 1 (P < 0.00001), I² = 98.4%								

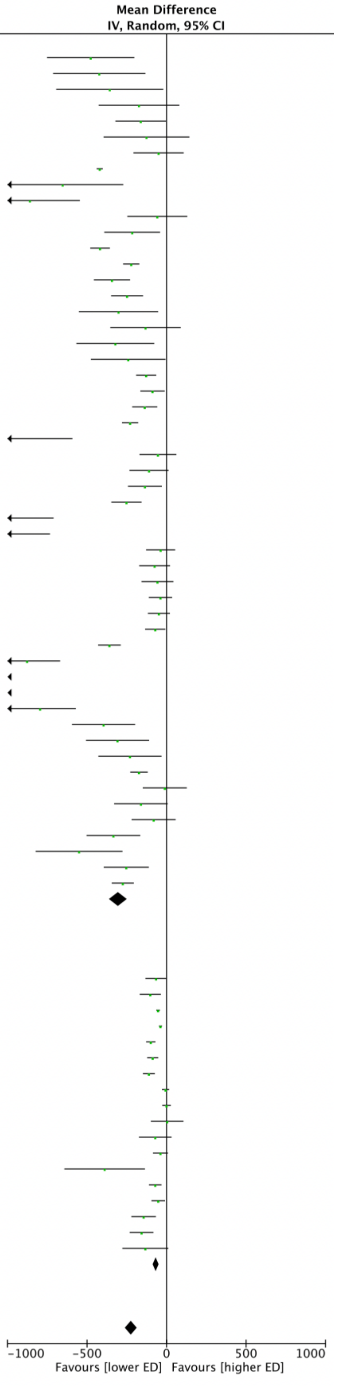

Figure S2 Quantitative analysis of the effects of meal type (preload versus entrée) on energy intake of randomized controlled crossover trials in humans receiving either lower energy density (ED) or higher ED diets

	lower ED			higher ED				Mean Difference
Study or Subgroup	Mean [kcal]	SD [kcal]	Total	Mean [kcal]	SD [kcal]	Total	Weight	IV, Random, 95% CI
**1.2.1 Preload design**								
Blatt et al. (2012): Energy100%; Men	2,664	482	28	2,837	487	28	1.0%	−173.00 [−426.80, 80.80]
Blatt et al. (2012): Energy100%; Women	1,907	367	40	2,069	361	40	1.4%	−162.00 [−321.53, −2.47]
Blatt et al. (2012): Energy64%; Men	2,392	471	28	2,518	556	28	0.9%	−126.00 [−395.90, 143.90]
Blatt et al. (2012): Energy64%; Women	1,780	379	40	1,830	342	40	1.4%	−50.00 [−208.20, 108.20]
Gray et al. (2003): 150ml	665	274	18	723	303	18	1.2%	−58.00 [−246.72, 130.72]
Gray et al. (2003): 450ml	713	280	18	929	257	18	1.3%	−216.00 [−391.58, −40.42]
Hogenkamp et al. (2010): Preload design	487	199	105	709	179	105	1.8%	−222.00 [−273.20, −170.80]
Kral et al. (2012): Normalweight	567	254	47	563	254	47	1.6%	4.00 [−98.69, 106.69]
Kral et al. (2012): Obesity	613	254	47	684	254	47	1.6%	−71.00 [−173.69, 31.69]
Kral et al. (2020)	618	248	212	656	248	212	1.8%	−38.00 [−85.21, 9.21]
Rolls et al. (1988): Orange jello	534	204	12	588	21	12	1.6%	−54.00 [−170.03, 62.03]
Rolls et al. (1988): Tomato soup	466	156	12	576	152	12	1.5%	−110.00 [−233.23, 13.23]
Rolls et al. (2004): 150g	786	243	42	922	255	42	1.6%	−136.00 [−242.53, −29.47]
Rolls et al. (2004): 300g	743	244	42	995	199	42	1.6%	−252.00 [−347.22, −156.78]
Yeomans & Chambers (2011): High Sensory	788	300	36	799	300	36	1.5%	−11.00 [−149.59, 127.59]
Yeomans & Chambers (2011): Low Sensory	768	420	36	929	300	36	1.3%	−161.00 [−329.60, 7.60]
Yeomans & Chambers (2011): Medium Sensory	748	300	36	829	300	36	1.5%	−81.00 [−219.59, 57.59]
**Subtotal (95% CI)**			**799**			**799**	**24.5%**	**−110.87 [−159.22, −62.52]**
Heterogeneity: $Tau^2 = 5767.59$; $Chi^2 = 47.52$, df = 16 (P < 0.0001); $I^2 = 66\%$								
Test for overall effect: Z = 4.49 (P < 0.00001)								
**1.2.2 Entrée design**								
Bell & Rolls (2001): Lean	1,868	432	19	2,345	432	19	0.9%	−477.00 [−751.71, −202.29]
Bell & Rolls (2001): Obese	1,729	432	17	2,152	432	17	0.9%	−423.00 [−713.42, −132.58]
Blatt et al. (2011)	2,760	704	41	3,117	845	41	0.7%	−357.00 [−693.65, −20.35]
Cheskin et al. (2008)	310	43	54	730	58	54	1.8%	−420.00 [−439.26, −400.74]
Devitt & Mattes (2004): Customary Food Unit	1,611	438	20	2,264	751	20	0.6%	−653.00 [−1034.02, −271.98]
Devitt & Mattes (2004): Small Food Unit	1,391	286	20	2,250	657	20	0.8%	−859.00 [−1173.04, −544.96]
Fisher et al. (2007): 250g	445	175	53	511	175	53	1.7%	−66.00 [−132.63, 0.63]
Fisher et al. (2007): 500g	496	175	53	598	175	53	1.7%	−102.00 [−168.63, −35.37]
Hogenkamp et al. (2010): Entreé design	249	126	105	667	298	105	1.7%	−418.00 [−479.88, −356.12]
Hogenkamp et al. (2012): Liquid food	132	82	27	475	291	27	1.6%	−343.00 [−457.04, −228.96]
Hogenkamp et al. (2012): Semi-solid food	92	78	27	340	255	27	1.6%	−248.00 [−348.58, −147.42]
Johnson et al. (1991): Experiment 1	59	9	12	112	18	12	1.8%	−53.00 [−64.39, −41.61]
Johnson et al. (1991): Experiment 2	31	5	9	69	14	9	1.8%	−38.00 [−47.71, −28.29]
Karl et al. (2013): Fast Eating Rate	697	336	20	999	459	20	1.0%	−302.00 [−551.30, −52.70]
Karl et al. (2013): Slow Eating Rate	601	283	20	733	419	20	1.1%	−132.00 [−353.59, 89.59]
Kling, Roe, Keller, et al. (2016): 100%	222	99	120	321	131	120	1.8%	−99.00 [−128.38, −69.62]
Kling, Roe, Keller, et al. (2016): 150%	279	121	120	366	153	120	1.8%	−87.00 [−121.90, −52.10]
Kling, Roe, Keller, et al. (2016): 200%	279	131	120	390	164	120	1.8%	−111.00 [−148.55, −73.45]
Kling, Roe, Sanchez, et al. (2016): 100%	322	88	125	328	97	125	1.8%	−6.00 [−28.96, 16.96]
Kling, Roe, Sanchez, et al. (2016): 150%	337	109	125	337	109	125	1.8%	0.00 [−27.02, 27.02]
Kral et al. (2002): Info	1,373	358	20	1,695	429	20	1.0%	−322.00 [−566.88, −77.12]
Kral et al. (2002): NoInfo	1,351	358	20	1,592	398	20	1.1%	−241.00 [−475.61, −6.39]
Kral et al. (2004): 500g	398	102	39	526	174	39	1.7%	−128.00 [−191.30, −64.70]
Kral et al. (2004): 700g	472	140	39	560	201	39	1.7%	−88.00 [−164.88, −11.12]
Kral et al. (2004): 900g	482	152	39	619	201	39	1.7%	−137.00 [−216.09, −57.91]
Leahy, Birch, Fisher et al. (2008)	2,350	347	26	2,739	561	26	1.0%	−389.00 [−642.55, −135.45]
Leahy, Birch & Rolls (2008): 300g	294	106	61	365	116	61	1.8%	−71.00 [−110.43, −31.57]
Leahy, Birch & Rolls (2008): 400g	311	115	61	363	126	61	1.8%	−52.00 [−94.81, −9.19]
McCrickerd et al. (2017)	286	76	58	515	183	58	1.8%	−229.00 [−280.00, −178.00]
Olsen et al. (2011)	209	179	74	353	288	74	1.7%	−144.00 [−221.26, −66.74]
Poppitt & Swann (1998)	2,054	693	5	3,535	741	5	0.2%	−1481.00 [−2370.28, −591.72]
Rolls et al. (2006): 100%	3,873	612	24	4,955	700	24	0.6%	−1082.00 [−1453.99, −710.01]
Rolls et al. (2006): 75%	3,355	558	24	4,449	710	24	0.7%	−1094.00 [−1455.28, −732.72]
Rolls et al. (2010): Addition study; 180g	555	224	49	592	238	49	1.7%	−37.00 [−128.51, 54.51]
Rolls et al. (2010): Addition study; 270g	541	245	49	616	245	49	1.6%	−75.00 [−172.01, 22.01]
Rolls et al. (2010): Addition study; 360g	574	238	49	631	268	49	1.6%	−57.00 [−157.36, 43.36]
Rolls et al. (2010): Substitution study; 180g	514	187	48	552	180	48	1.7%	−38.00 [−111.43, 35.43]
Rolls et al. (2010): Substitution study; 270g	469	166	48	517	180	48	1.7%	−48.00 [−117.27, 21.27]
Rolls et al. (2010): Substitution study; 360g	466	152	48	537	166	48	1.7%	−71.00 [−134.67, −7.33]
Silver et al. (2008)	415	104	45	774	221	45	1.7%	−359.00 [−430.36, −287.64]
Smethers et al. (2019)	884	266	49	1,041	21	49	1.7%	−157.00 [−231.71, −82.29]
Spill et al. (2011)	890	325	39	1,023	327	39	1.4%	−133.00 [−277.69, 11.69]
Stubbs, Harbron et al. (1995)	2,176	146	7	3,053	240	7	1.2%	−877.00 [−1085.10, −668.90]
Stubbs, Johnstone, Harbron et al. (1998)	2,072	129	6	3,540	222	6	1.2%	−1468.00 [−1673.45, −1262.55]
Stubbs, Johnstone, O'Reilly et al. (1998)	2,434	240	6	3,862	351	6	0.7%	−1428.00 [−1768.23, −1087.77]
Stubbs, Ritz et al. (1995)	2,157	146	6	2,952	240	6	1.1%	−795.00 [−1019.78, −570.22]
Williams et al. (2013): Fat manipulation	2,272	507	59	2,668	591	59	1.2%	−396.00 [−594.69, −197.31]
Williams et al. (2013): Fruits/Veggie manipulation	2,360	507	59	2,668	591	59	1.2%	−308.00 [−506.69, −109.31]
Williams et al. (2013): Water manipulation	2,438	507	59	2,668	591	59	1.2%	−230.00 [−428.69, −31.31]
Williams et al. (2014)	476	109	46	649	156	46	1.8%	−173.00 [−228.00, −118.00]
Yeomans et al. (2005): Experiment 1; bland	359	191	16	693	287	16	1.3%	−334.00 [−502.92, −165.08]
Yeomans et al. (2005): Experiment 1; palatable	382	287	16	932	478	16	0.9%	−550.00 [−823.19, −276.81]
Yeomans et al. (2005): Experiment 2	177	38	16	430	287	16	1.4%	−253.00 [−394.85, −111.15]
Yeomans et al. (2009)	174	46	12	449	114	12	1.7%	−275.00 [−344.55, −205.45]
**Subtotal (95% CI)**			**2329**			**2329**	**75.5%**	**−261.23 [−304.58, −217.88]**
Heterogeneity: $Tau^2 = 20277.81$; $Chi^2 = 2132.46$, df = 53 (P < 0.00001); $I^2 = 98\%$								
Test for overall effect: Z = 11.81 (P < 0.00001)								
**Total (95% CI)**			**3128**			**3128**	**100.0%**	**−222.85 [−259.67, −186.04]**
Heterogeneity: $Tau^2 = 19199.97$; $Chi^2 = 2181.86$, df = 70 (P < 0.00001); $I^2 = 97\%$								
Test for overall effect: Z = 11.86 (P < 0.00001)								
Test for subgroup differences: $Chi^2 = 20.60$, df = 1 (P < 0.00001), $I^2 = 95.1\%$								

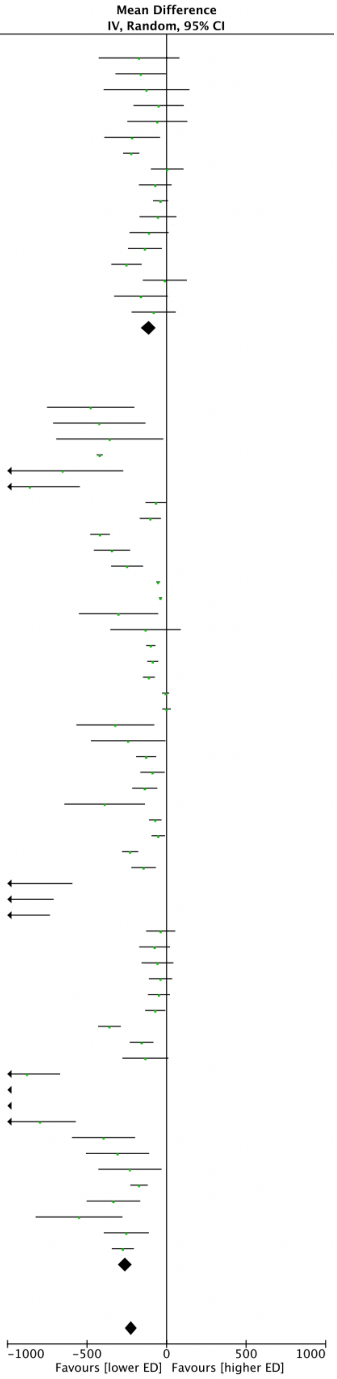

Figure S3 Quantitative analysis of the effects of intervention length (1 meal intervention versus > 1 meal intervention) on energy intake of randomized controlled crossover trials in humans receiving either lower energy density (ED) or higher ED diets

	lower ED			higher ED				Mean Difference
**Study or Subgroup**	**Mean [kcal]**	**SD [kcal]**	**Total**	**Mean [kcal]**	**SD [kcal]**	**Total**	**Weight**	**IV, Random, 95% CI**
**1.3.1 1 Meal Intervention**								
Cheskin et al. (2008)	310	43	54	730	58	54	1.8%	−420.00 [−439.26, −400.74]
Fisher et al. (2007): 250g	445	175	53	511	175	53	1.7%	−66.00 [−132.63, 0.63]
Fisher et al. (2007): 500g	496	175	53	598	175	53	1.7%	−102.00 [−168.63, −35.37]
Gray et al. (2003): 150ml	665	274	18	723	303	18	1.2%	−58.00 [−246.72, 130.72]
Gray et al. (2003): 450ml	713	280	18	929	257	18	1.3%	−216.00 [−391.58, −40.42]
Hogenkamp et al. (2010): Entreé design	249	126	105	667	298	105	1.7%	−418.00 [−479.88, −356.12]
Hogenkamp et al. (2010): Preload design	487	199	105	709	179	105	1.8%	−222.00 [−273.20, −170.80]
Hogenkamp et al. (2012): Liquid food	132	82	27	475	291	27	1.6%	−343.00 [−457.04, −228.96]
Hogenkamp et al. (2012): Semi-solid food	92	78	27	340	255	27	1.6%	−248.00 [−348.58, −147.42]
Johnson et al. (1991): Experiment 1	59	9	12	112	18	12	1.8%	−53.00 [−64.39, −41.61]
Johnson et al. (1991): Experiment 2	31	5	9	69	14	9	1.8%	−38.00 [−47.71, −28.29]
Karl et al. (2013): Fast Eating Rate	697	336	20	999	459	20	1.0%	−302.00 [−551.30, −52.70]
Karl et al. (2013): Slow Eating Rate	601	283	20	733	419	20	1.1%	−132.00 [−353.59, 89.59]
Kling, Roe, Keller, et al. (2016): 100%	222	99	120	321	131	120	1.8%	−99.00 [−128.38, −69.62]
Kling, Roe, Keller, et al. (2016): 150%	279	121	120	366	153	120	1.8%	−87.00 [−121.90, −52.10]
Kling, Roe, Keller, et al. (2016): 200%	279	131	120	390	164	120	1.8%	−111.00 [−148.55, −73.45]
Kling, Roe, Sanchez, et al. (2016): 100%	322	88	125	328	97	125	1.8%	−6.00 [−28.96, 16.96]
Kling, Roe, Sanchez, et al. (2016): 150%	337	109	125	337	109	125	1.8%	0.00 [−27.02, 27.02]
Kral et al. (2004): 500g	398	102	39	526	174	39	1.7%	−128.00 [−191.30, −64.70]
Kral et al. (2004): 700g	472	140	39	560	201	39	1.7%	−88.00 [−164.88, −11.12]
Kral et al. (2004): 900g	482	152	39	619	201	39	1.7%	−137.00 [−216.09, −57.91]
Kral et al. (2012): Normalweight	567	254	47	563	254	47	1.6%	4.00 [−98.69, 106.69]
Kral et al. (2012): Obesity	613	254	47	684	254	47	1.6%	−71.00 [−173.69, 31.69]
Kral et al. (2020)	618	248	212	656	248	212	1.8%	−38.00 [−85.21, 9.21]
Leahy, Birch & Rolls (2008): 300g	294	106	61	365	116	61	1.8%	−71.00 [−110.43, −31.57]
Leahy, Birch & Rolls (2008): 400g	311	115	61	363	126	61	1.8%	−52.00 [−94.81, −9.19]
McCrickerd et al. (2017)	286	76	58	515	183	58	1.8%	−229.00 [−280.00, −178.00]
Olsen et al. (2011)	209	179	74	353	288	74	1.7%	−144.00 [−221.26, −66.74]
Rolls et al. (1988): Orange jello	534	204	12	588	21	12	1.6%	−54.00 [−170.03, 62.03]
Rolls et al. (1988): Tomato soup	466	156	12	576	152	12	1.5%	−110.00 [−233.23, 13.23]
Rolls et al. (2004): 150g	786	243	42	922	255	42	1.6%	−136.00 [−242.53, −29.47]
Rolls et al. (2004): 300g	743	244	42	995	199	42	1.6%	−252.00 [−347.22, −156.78]
Rolls et al. (2010): Addition study; 180g	555	224	49	592	238	49	1.7%	−37.00 [−128.51, 54.51]
Rolls et al. (2010): Addition study; 270g	541	245	49	616	245	49	1.6%	−75.00 [−172.01, 22.01]
Rolls et al. (2010): Addition study; 360g	574	238	49	631	268	49	1.6%	−57.00 [−157.36, 43.36]
Rolls et al. (2010): Substitution study; 180g	514	187	48	552	180	48	1.7%	−38.00 [−111.43, 35.43]
Rolls et al. (2010): Substitution study; 270g	469	166	48	517	180	48	1.7%	−48.00 [−117.27, 21.27]
Rolls et al. (2010): Substitution study; 360g	466	152	48	537	166	48	1.7%	−71.00 [−134.67, −7.33]
Silver et al. (2008)	415	104	45	774	221	45	1.7%	−359.00 [−430.36, −287.64]
Williams et al. (2014)	476	109	46	649	156	46	1.8%	−173.00 [−228.00, −118.00]
Yeomans & Chambers (2011): High Sensory	788	300	36	799	300	36	1.5%	−11.00 [−149.59, 127.59]
Yeomans & Chambers (2011): Low Sensory	768	420	36	929	300	36	1.3%	−161.00 [−329.60, 7.60]
Yeomans & Chambers (2011): Medium Sensory	748	300	36	829	300	36	1.5%	−81.00 [−219.59, 57.59]
Yeomans et al. (2005): Experiment 1; bland	359	191	16	693	287	16	1.3%	−334.00 [−502.92, −165.08]
Yeomans et al. (2005): Experiment 1; palatable	382	287	16	932	478	16	0.9%	−550.00 [−823.19, −276.81]
Yeomans et al. (2005): Experiment 2	177	38	16	430	287	16	1.4%	−253.00 [−394.85, −111.15]
Yeomans et al. (2009)	174	46	12	449	114	12	1.7%	−275.00 [−344.55, −205.45]
**Subtotal (95% CI)**			**2466**			**2466**	**76.1%**	**−140.73 [−179.21, −102.25]**
Heterogeneity: $Tau^2 = 15600.27$; $Chi^2 = 1719.82$, df = 46 (P < 0.00001); $I^2 = 97\%$								
Test for overall effect: Z = 7.17 (P < 0.00001)								
**1.3.2 > 1 Meal Intervention**								
Bell & Rolls (2001): Lean	1,868	432	19	2,345	432	19	0.9%	−477.00 [−751.71, −202.29]
Bell & Rolls (2001): Obese	1,729	432	17	2,152	432	17	0.9%	−423.00 [−713.42, −132.58]
Blatt et al. (2011)	2,760	704	41	3,117	845	41	0.7%	−357.00 [−693.65, −20.35]
Blatt et al. (2012): Energy100%; Men	2,664	482	28	2,837	487	28	1.0%	−173.00 [−426.80, 80.80]
Blatt et al. (2012): Energy100%; Women	1,907	367	40	2,069	361	40	1.4%	−162.00 [−321.53, −2.47]
Blatt et al. (2012): Energy64%; Men	2,392	471	28	2,518	556	28	0.9%	−126.00 [−395.90, 143.90]
Blatt et al. (2012): Energy64%; Women	1,780	379	40	1,830	342	40	1.4%	−50.00 [−208.20, 108.20]
Devitt & Mattes (2004): Customary Food Unit	1,611	438	20	2,264	751	20	0.6%	−653.00 [−1034.02, −271.98]
Devitt & Mattes (2004): Small Food Unit	1,391	286	20	2,250	657	20	0.8%	−859.00 [−1173.04, −544.96]
Kral et al. (2002): Info	1,373	358	20	1,695	429	20	1.0%	−322.00 [−566.88, −77.12]
Kral et al. (2002): NoInfo	1,351	358	20	1,592	398	20	1.1%	−241.00 [−475.61, −6.39]
Leahy, Birch, Fisher et al. (2008)	2,350	347	26	2,739	561	26	1.0%	−389.00 [−642.55, −135.45]
Poppitt & Swann (1998)	2,054	693	5	3,535	741	5	0.2%	−1481.00 [−2370.28, −591.72]
Rolls et al. (2006): 100%	3,873	612	24	4,955	700	24	0.6%	−1082.00 [−1453.99, −710.01]
Rolls et al. (2006): 75%	3,355	558	24	4,449	710	24	0.7%	−1094.00 [−1455.28, −732.72]
Smethers et al. (2019)	884	266	49	1,041	21	49	1.7%	−157.00 [−231.71, −82.29]
Spill et al. (2011)	890	325	39	1,023	327	39	1.4%	−133.00 [−277.69, 11.69]
Stubbs, Harbron et al. (1995)	2,176	146	7	3,053	240	7	1.2%	−877.00 [−1085.10, −668.90]
Stubbs, Johnstone, Harbron et al. (1998)	2,072	129	6	3,540	222	6	1.2%	−1468.00 [−1673.45, −1262.55]
Stubbs, Johnstone, O'Reilly et al. (1998)	2,434	240	6	3,862	351	6	0.7%	−1428.00 [−1768.23, −1087.77]
Stubbs, Ritz et al. (1995)	2,157	146	6	2,952	240	6	1.1%	−795.00 [−1019.78, −570.22]
Williams et al. (2013): Fat manipulation	2,272	507	59	2,668	591	59	1.2%	−396.00 [−594.69, −197.31]
Williams et al. (2013): Fruits/Veggie manipulation	2,360	507	59	2,668	591	59	1.2%	−308.00 [−506.69, −109.31]
Williams et al. (2013): Water manipulation	2,438	507	59	2,668	591	59	1.2%	−230.00 [−428.69, −31.31]
**Subtotal (95% CI)**			**662**			**662**	**23.9%**	**−535.32 [−694.11, −376.53]**
Heterogeneity: $Tau^2 = 137010.25$; $Chi^2 = 303.56$, df = 23 (P < 0.00001); $I^2 = 92\%$								
Test for overall effect: Z = 6.61 (P < 0.00001)								
**Total (95% CI)**			**3128**			**3128**	**100.0%**	**−222.85 [−259.67, −186.04]**
Heterogeneity: $Tau^2 = 19199.97$; $Chi^2 = 2181.86$, df = 70 (P < 0.00001); $I^2 = 97\%$								
Test for overall effect: Z = 11.86 (P < 0.00001)								
Test for subgroup differences: $Chi^2 = 22.41$, df = 1 (P < 0.00001), $I^2 = 95.5\%$								

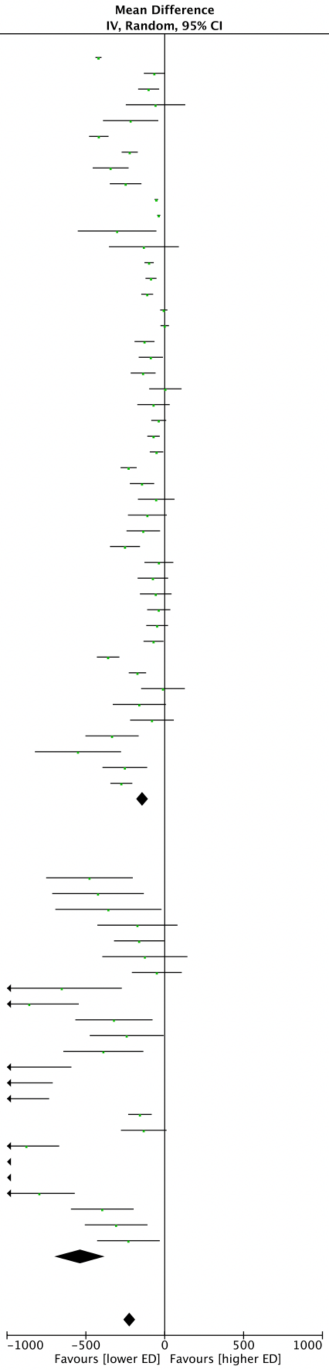

Supplement: Supplementary file 1 — Supplementary file1 (PDF 4332 KB) [file 394_2022_3054_MOESM1_ESM.pdf]
